# Atomic Nb-doping of WS_2_ for high-performance synaptic transistors in neuromorphic computing

**DOI:** 10.1038/s41378-024-00779-1

**Published:** 2024-09-26

**Authors:** Kejie Guan, Yinxiao Li, Lin Liu, Fuqin Sun, Yingyi Wang, Zhuo Zheng, Weifan Zhou, Cheng Zhang, Zhengyang Cai, Xiaowei Wang, Simin Feng, Ting Zhang

**Affiliations:** 1 i-Lab, Suzhou Institute of Nano-Tech and Nano-Bionics (SINANO), Chinese Academy of Sciences (CAS), Suzhou, Jiangsu 215123 China; 2 School of Nano-Tech and Nano-Bionics, University of Science and Technology of China, Hefei, Anhui 230026 China; 3 School of Electronic and Optical Engineering, Nanjing University of Science and Technology, Nanjing, Jiangsu 210094 China; 4 Department of Health and Environmental Sciences, Xi’an Jiaotong-Liverpool University, 111 Renai Road, Suzhou, Jiangsu 215123 China; 5 Jiangsu Key Laboratory of Micro and Nano Heat Fluid Flow Technology and Energy Application, School of Physical Science and Technology, Suzhou University of Science and Technology, Suzhou, Jiangsu 215009 China; 6 Department of Electronic Engineering, Jiangnan University, Wuxi, Jiangsu 214122 China; 7 Nano-X Vacuum Interconnected Workstation, Suzhou Institute of Nano-Tech & Nano-Bionics (SINANO), Chinese Academy of Sciences (CAS), Suzhou, Jiangsu 215123 China

**Keywords:** Electronic properties and materials, Electronic devices

## Abstract

Owing to the controllable growth and large-area synthesis for high-density integration, interest in employing atomically thin two-dimensional (2D) transition-metal dichalcogenides (TMDCs) for synaptic transistors is increasing. In particular, substitutional doping of 2D materials allows flexible modulation of material physical properties, facilitating precise control in defect engineering for eventual synaptic plasticity. In this study, to increase the switch ratio of synaptic transistors, we selectively performed experiments on WS_2_ and introduced niobium (Nb) atoms to serve as the channel material. The Nb atoms were substitutionally doped at the W sites, forming a uniform distribution across the entire flakes. The synaptic transistor devices exhibited an improved switch ratio of 10^3^, 100 times larger than that of devices prepared with undoped WS_2_. The Nb atoms in WS_2_ play crucial roles in trapping and detrapping electrons. The modulation of channel conductivity achieved through the gate effectively simulates synaptic potentiation, inhibition, and repetitive learning processes. The Nb-WS_2_ synaptic transistor achieves 92.30% recognition accuracy on the Modified National Institute of Standards and Technology (MNIST) handwritten digit dataset after 125 training iterations. This study’s contribution extends to a pragmatic and accessible atomic doping methodology, elucidating the strategies underlying doping techniques for channel materials in synaptic transistors.

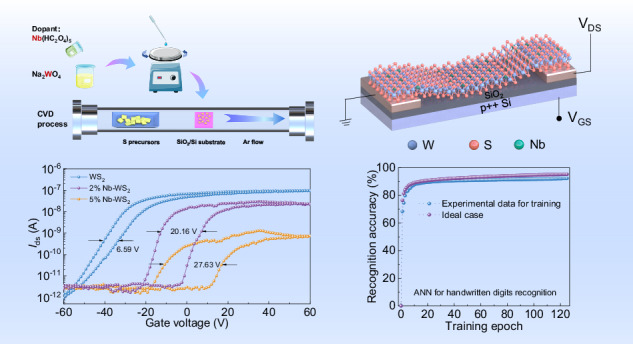

## Introduction

To overcome the inherent power wall of von Neumann architectures, there is an increasing emphasis on neuromorphic computing techniques that emulate the energy-efficient information processing observed in biological neural networks^1–6^. Memristive switching devices, considered for the next generation of digital memory, offer unique properties such as high switching speeds, high scalability, and high integration density^7,8^. Notably, the conductance of memristive devices can be gradually modulated by external electrical pulses, akin to the updating of synaptic weights in biological synapses^9–13^. In contrast to conventional metal oxides used for memristive devices, atomically thin two-dimensional (2D) materials offer advantages such as power efficiency, reliability, tunability, heterointegration, and multifunctionality, which are essential for nonvolatile memory (NVM) devices^14,15^.

In the current research, 2D materials employ mechanisms such as grain boundary-induced vacancies^16–18^, phase transitions^19–21^, and Schottky barriers^22–24^ to achieve memory functions in three-terminal NVM devices. However, the characteristics of memristors based on these mechanisms primarily depend on the intrinsic quality and manufacturing processes of the active material. Two-dimensional materials that have been previously reported often exhibit uncontrollable material properties, including grain boundary density and grain size^25,26^. In addition to the abovementioned methods, substitutional doping is another controllable and efficient approach for modifying materials to achieve resistance-switching devices. This process enables the precise tuning of electronic properties, imparting rich physical functionality^27,28^. In memristive devices employing doped 2D materials, the concentration of electrons in the material is modulated through significant voltage tunability, influencing conductance via the capture and release of electrons at defect centers to achieve high performance. For example, Appenzeller et al. reported an electric-field-induced structural transition in 2H-MoTe_2_- and Mo_1−x_W_x_Te_2_-based resistive random-access memory (RRAM) devices that achieved on/off current ratios of 10^6^ and low programming currents in a selectorless RRAM architecture via an Al_2_O_3_/MoTe_2_ stack^29^. Wang et al. created MoS_2–x_O_x_ via O_2_ dry etching in a plasma system and designed a graphene/MoS_2–x_O_x_/graphene heterostructure that exhibited excellent switching performance with an endurance of up to 10^7^ ^30^. Additionally, Cheng et al. fabricated artificial synaptic transistors using heavily V-doped MoS_2_ as the channel material, demonstrating synaptic potentiation, depression, and repetitive learning processes through gate-tunable changes in channel conductance with abundant vanadium atoms trapping/detrapping electrons^31^.

The role of atomic doping in Transition Metal Dichalcogenide (TMDC) materials in the memristor mechanism of devices is unclear, with the ongoing debate being whether the trapping/detrapping mechanism of doped atoms or the impact of substrate oxide defects plays a more significant role in synaptic transistors. Clarifying the effect of dopant atoms in alternative doping methods is essential for optimizing neuromorphic devices based on TMDCs. In this work, we propose a p-type doping strategy in WS_2_ to increase the conductance ratio and improve the linearity of weight updates in synaptic transistors. Using liquid-phase precursor chemical vapor deposition (CVD), we achieved uniform incorporation of Nb atoms into WS_2_ crystals. Characterization techniques, including scanning transmission electron microscopy (STEM), Raman spectroscopy, photoluminescence (PL), and X-ray photoelectron spectroscopy (XPS), confirmed doping-induced alterations in the atomic structure. The synaptic transistor, which was fabricated with a monolayer of Nb-doped WS_2_ as the channel material, demonstrated an enhanced switching ratio, surpassing that of undoped WS_2_ by a factor of 100. When applied to the recognition of the Modified National Institute of Standards and Technology (MNIST) handwritten digit sets, the synaptic device achieved an accuracy of 92.30%.

## Results and Discussion

### WS_2_ monolayer flake growth and Nb atom substitutional doping

**Fig. 1:**
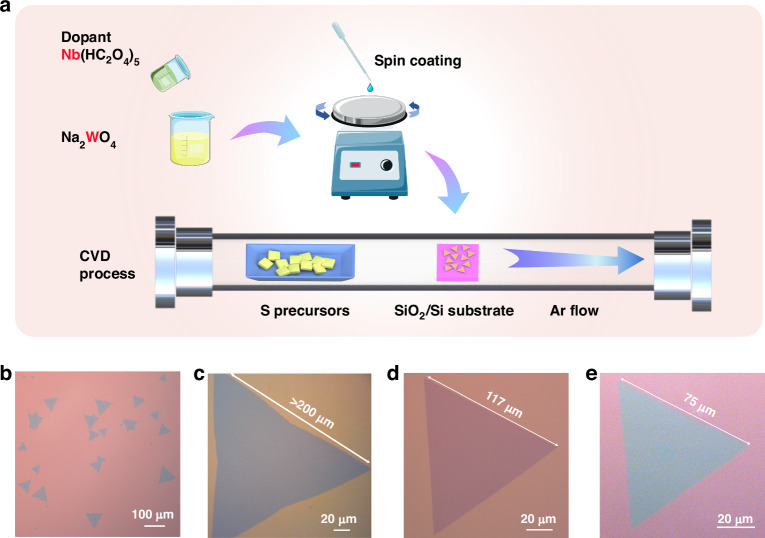
Material growth and sample photomicrograph. **a** Illustration of the CVD growth process for both doped and undoped WS_2_. **b** Low-magnification optical image revealing undoped WS_2_ with uniformly distributed flakes on the growth substrate. **c-e** Optical images of undoped WS_2_, 2% Nb-doped WS_2_, and 5% Nb-doped WS_2_, respectively

The growth of Nb-doped WS_2_ layers was conducted through atmospheric pressure CVD, as outlined in Fig. 1a. A precursor solution with a concentration of 1.8 mg/mL was created by combining niobium oxalate (Nb(HC_2_O_4_)_5_·xH_2_O) with sodium tungstate (Na_2_WO_4_·2H_2_O). To prepare Nb-WS_2_ with nominal doping concentrations of 0% (undoped), 2%, and 5%, we precisely controlled the ratio of W and Nb atoms in the precursor reagent. The prepared materials and devices were named based on the precursor concentrations, 2% Nb-WS_2_ and 5% Nb-WS_2_, but these designations do not represent their actual doping concentrations. After the SiO_2_/Si substrate was spin-coated with the precursor solution, the substrate was placed in a one-inch quartz tube and heated to 875 °C. Monolayer WS_2_ crystallites with triangular morphologies were obtained by adjusting the hold duration and Ar gas flow rate. After a 15-minute growth period and natural cooling, WS_2_ monolayer flakes were uniformly distributed across the entire silicon wafer, as shown in Fig. 1b. Pristine WS_2_ yielded equilateral triangles with side lengths exceeding 200 μm (Fig. 1c). In comparison, the maximum lateral dimensions of the thin films with 2% Nb dopant and 5% Nb dopant added reached 117 μm and 75 μm, respectively (Fig. 1d, e). We systematically examined 100 triangular flakes across three different doping concentrations and recorded their dimensions. The findings revealed that the average sizes of WS_2_, 2% Nb-WS_2_, and 5% Nb-WS_2_ were 125.3 μm, 62.5 μm, and 31.6 μm, respectively (Fig. S1). Interestingly, as the doping concentration increased, both the average and maximum edge lengths noticeably decreased. This trend suggests a correlation between the doping level and the geometric properties of the Nb-WS_2_ flakes, highlighting the material’s sensitivity to dopant-induced structural changes. The difference in lateral dimensions may be attributed to the absence of impurity atoms, resulting in minimal atomic distortion and facilitating the formation of larger flakes. The thickness of the CVD-grown WS_2_ was assessed via atomic force microscopy (AFM), revealing a typical monolayer flake with a height of 0.86 nm (Fig. S2).

### Characterizing the crystal structure changes induced by doping

**Fig. 2:**
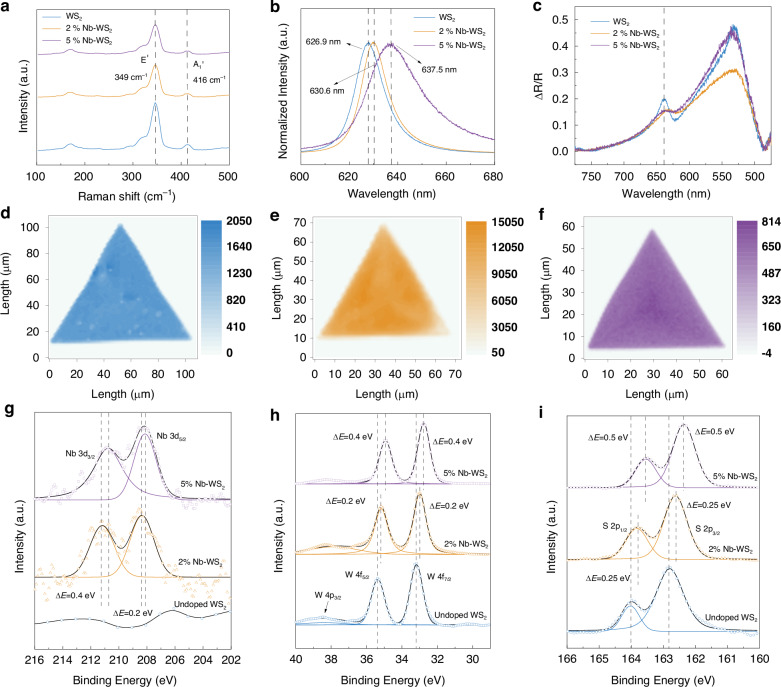
Characterization of p-type doping and homogeneity of CVD-grown Nb-WS_2_. **a** Raman spectra of Nb-WS_2_, exhibiting peaks at 349 cm^−1^ and 416 cm^−1^. **b** PL spectra of Nb-WS_2,_ revealing emission characteristics at approximately 630 nm. The emission wavelength of undoped WS_2_ is 626.9 nm, whereas the emission wavelengths of 2% and 5% Nb-doped WS_2_ redshifted by 3.7 nm and 10.6 nm, respectively. **c** Differential reflectivity of CVD Nb-WS_2_ flakes. The neutral exciton peak was observed at 630 nm. **d-f** Raman mapping graphs illustrating the high homogeneity of undoped WS_2_, 2% Nb-WS_2_, and 5% Nb-WS_2_. XPS spectra and curve fitting of **g** Nb, **h** W, and **i** S

To evaluate the quality of the grown WS_2_ flakes, we employed a comprehensive characterization approach involving Raman, photoluminescence (PL), and XPS measurements. In the Raman spectra, characteristic vibration peaks of E’ and A_1_’ are observed at 349 cm^−1^ and 416 cm^−1^, aligning with the distinctive monolayer WS_2_ peaks (Fig. 2a)^32,33^. The normalized PL spectra of WS_2_ with various doping concentrations are depicted in Fig. 2b. The emission position redshifted from 626.9 nm to 637.5 nm with increasing Nb ratio up to 5%, which is indicative of p-type doping induced by Nb. The reflection contrast spectrum (R_sample_-R_sub_)/R_sub_ was used to characterize the synthesized Nb-WS_2_. Here, R_sample_ represents the reflectivity of monolayer WS_2_ on a SiO_2_/Si substrate with a thickness of 285 nm, whereas R_sub_ corresponds to the reflectivity of the substrate. The spectrum indicates easy excitation of photons within the material at wavelengths between 630 and 640 nm (Fig. 2c), which is in good agreement with the PL peaks. The broad peak at approximately 530 nm is attributed to the gradually changing background, which is primarily induced by the 285 nm-thick SiO_2_/Si substrate^34^. These results confirm the successful growth of monolayer Nb-doped WS_2_. Figure 2d–f displays Raman mapping images of WS_2_ with different doping concentrations. The well-defined and homogeneous emission patterns across all the pristine and doped WS_2_ samples are illustrated in the Raman mapping results. These graphs provide evidence of the excellent Raman properties of Nb-WS_2_, emphasizing its uniform spatial distribution.

Figure 2g–i present high-resolution XPS spectra of Nb, W, and S in the as-grown flakes. As shown in Fig. 2g, no Nb peak was discernible in the XPS pattern of undoped WS_2_. In contrast, both the Nb 3d_3/2_ and Nb 3d_5/2_ peaks were observed for the 2% and 5% doped WS_2_ samples. Notably, the peaks in the plots redshifted for both W and S due to atom doping. Compared with those of undoped WS_2_, the W 4f_5/2_ and W 4f_7/2_ orbitals of 2% Nb-doped WS_2_ redshifted by 0.2 eV (Table S1). Moreover, the S 2p_1/2_ and S 2p_3/2_ orbitals exhibited a redshift of 0.25 eV. These shifts suggested that the bonding states of some W and S atoms were changed by the nearby substituted Nb atoms. Compared with those of 2% Nb-WS_2_, the binding energies of the Nb 3d_3/2_ peak and Nb 3d_5/2_ peak observed for 5% Nb-WS_2_ redshifted by 0.4 eV and 0.2 eV, respectively. Similarly, the binding energies of W and S in the 5% Nb-doped WS_2_ sample further redshifted from those of the 2% Nb-WS_2_ sample. These shifts in the lower binding energy direction indicated a shift in the Fermi level (E_F_) of WS_2_ toward the valence band upon increasing the doping concentration, thereby confirming the successful attainment of p-type doping through the introduction of Nb^35^. On the basis of the results of the PL spectra and XPS measurements, the impact of atom doping on the electronic structure of WS_2_ is illustrated in Fig. S3a. Upon p-doping, the valence band maximum shifts closer to E_F_^36^. The energy difference between the valence band maximum and the E_F_ decreases with increasing doping concentration. This upward shift in the valence and conduction bands is further supported by density functional theory (DFT) calculations (Fig. S3b, c). In addition, the presence of defects induced by Nb doping in the material was confirmed through in situ low-temperature PL analysis. Fig. S4 presents a typical curve fit of the 5% Nb-WS_2_ PL spectrum at 100 K. The introduction of Nb atoms increases the number of defects in the material, leading to the detection of the characteristic X_B_ peak of defects, located at ~1.88 eV^37^. The results of the above assessments confirmed the successful incorporation of Nb into the WS_2_ film via the addition of niobium oxalate to the precursor, effectively achieving p-type doping. As the doping concentration increased, the peak positions for Nb, W, and S all redshifted, indicating the occurrence of p-type doping subsequent to the introduction of Nb. Additionally, an increased doping concentration correlates with an increase in hole concentration.

**Fig. 3:**
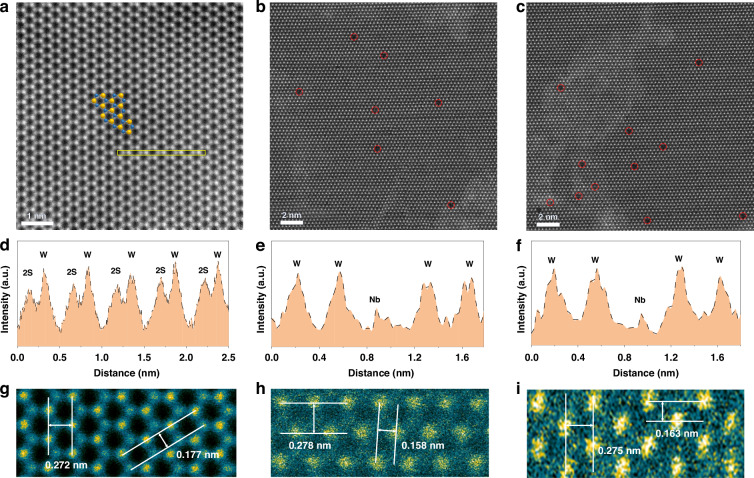
TEM image of a monolayer WS_2_ lattice substitutionally doped with Nb. **a** Aberration-corrected Z-contrast STEM image of monolayer undoped WS_2_. **b-c** STEM images of monolayer 2% and 5% Nb-WS_2_, respectively. The intensity profile of the brightness of atoms, marked by yellow rectangular regions in Panels **a**, **b**, and **c**, is displayed in Panels **d,** **e**, and **f**, respectively. **g**–**i** Magnified STEM images of typical regions. The yellow sites represent tungsten atoms, whereas the green sites represent sulfur atoms

The lattice structure of Nb-WS_2_ was systematically characterized via spherical aberration-corrected transmission electron microscopy (TEM). The transferred flakes exhibit excellent thickness uniformity, which is consistent with the monolayer observed in the AFM results (Fig. S5a). High-angle annular dark-field scanning transmission electron microscopy (HAADF-STEM) images showed distinct brightness contrast for W, S, and Nb atoms, with the contrast directly correlating to the atomic number of the elements. In undoped WS_2_, a flawless six-membered ring lattice was evident, as illustrated in the pseudocolor image (Fig. 3a). The intensity in the annular dark-field (ADF) image directly corresponded to the atomic number, enabling spatially resolved chemical identification through quantitative analysis of image intensity. Brighter atoms (W) exhibited a contrasting hue, whereas darker atoms (S) manifested as different shades in the pseudocolor representation, displayed as yellow and blue, respectively. The selected area electron diffraction (SAED) pattern exhibited a hexagonal structure with threefold symmetry, featuring two sets of spots, *K*_a_ (indicated by blue arrows) and *K*_b_ (indicated by red arrows) (Fig. S5b)^38^. Upon the incorporation of niobium oxalate into the precursor, the presence of darker atoms indicative of Nb was revealed, as shown in Fig. 3b, c and validated by the intensity profile analysis in Fig. 3d–f. On the basis of atomic counts, films synthesized from precursors with a nominal Nb content of 2% presented a measured doping concentration of approximately 0.2%. Conversely, films synthesized from 5% Nb precursors presented a relatively high doping concentration of approximately 0.3%. The atomic pseudocolor plots in Fig. 3g–i feature labeled crystal plane spacings of Nb-WS_2_. Notably, the crystal plane spacing of the (10-10) crystal plane is approximately 0.27 nm, whereas the range of crystal plane spacings for the (11–20) crystal plane is 0.16–0.18 nm, which is consistent with previously reported data^39,40^. The lattice parameters suggest that the introduction of Nb atoms as dopants did not induce alterations in the lattice structure of the WS_2_ flakes.

### Performance of Nb-WS_2_ synaptic transistors and their suitability for neuromorphic computing

**Fig. 4:**
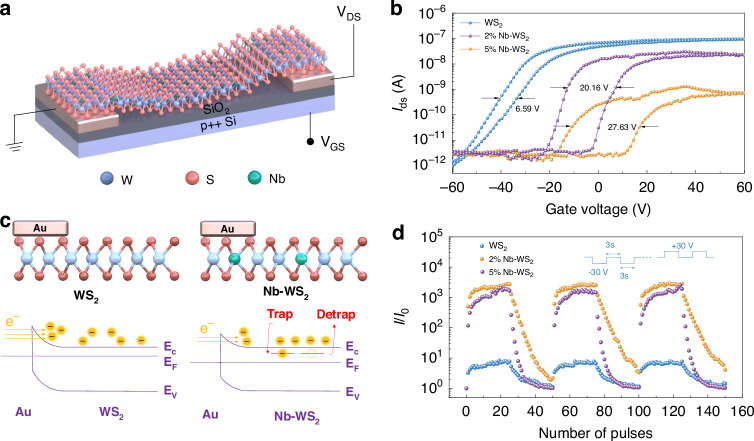
FET performance when undoped WS_2_ and Nb-WS_2_ are used as channel materials. **a** Schematic of the Nb-WS_2_-based three-terminal device built on a 285 nm SiO_2_/Si substrate: Nb-WS_2_ as a semiconducting channel, a 285 nm SiO_2_ layer as a dielectric layer and p^++^ Si as a back gate. Bias voltages were applied on the drain electrode and back gate, with the source electrode being grounded. **b** Transfer characteristics of the Nb-WS_2_-based device at V_ds_ = 0.5 V, showing different shifts and hysteresis in the threshold voltage, indicating that hysteresis can be engineered by changing the doping concentration of Nb atoms. **c** Schematic diagram of the causes of hysteresis. **d** Modulation of conductance under the continuous application of potentiation pulses (25 pulses of −30 V) and depression pulses (25 pulses of +30 V). The inset is a diagram of the applied pulse

We subsequently employed p-type doped materials in the fabrication of artificial synaptic transistors. As depicted in Fig. 4a, monolayer Nb-WS_2_ served as a channel material and was transferred onto predeposited metal electrodes through a wet etch transfer approach (refer to the Experimental section and Fig. S6 for details and Fig. S7 for typical optical images of the devices). The devices were constructed on a 285 nm SiO_2_/Si substrate, which functions as the back gate. In Fig. 4b, the transfer curves (I_ds_-V_gs_) of devices based on undoped WS_2_, 2% Nb-WS_2_ and 5% Nb-WS_2_ exhibit a continuous shift in the threshold voltage toward a positive voltage with increasing doping concentration. This observation reaffirms the enhancement of p-type transport behavior, validating the efficacy of the doping process. An on/off current ratio of 10^5^ is achieved for the WS_2_ field effect transistor (FET) within a sweeping range of 60 V. The switching ratios of the 2% Nb-WS_2_ and 5% Nb-WS_2_ FETs are only 10^4^ and 10^3^, respectively. Notably, varying the channel length due to the triangular shape of the Nb-WS_2_ flakes may influence the on-state and off-state currents (Fig. S8). However, the geometric parameters have little effect on the on/off ratio. We also investigated the electrical properties of Nb-WS_2_ films with a precursor Nb doping concentration of 7%. Our findings revealed a rapid decline in electrical and synaptic performance as the doping concentration increased (Fig. S9). Figure 4c illustrates the potential reason for the decreased on-current caused by defects attributed to Nb atom doping^41–43^. Hot electrons from the metal electrode surmount the effective Schottky barrier in the WS_2_ FET and transfer to the mobile state of the conduction band in WS_2_. Compared with that in the WS_2_ FET, the downward shift of the channel layer’s E_F_ in the Nb-WS_2_ FET leads to a flatter band at the Schottky barrier, increasing the effective width of the Schottky barrier and reducing the injection of hot electrons from the electrode. When V_gs_ > 0 V, the introduction of defects caused by doping increases carrier scattering, thereby reducing electron mobility. Importantly, all the transfer characteristic curves demonstrate substantial hysteresis windows. While pristine WS_2_ has a hysteresis window of only 6.59 V, 2% and 5% Nb-WS_2_ have hysteresis windows of 20.16 V and 27.63 V, respectively. Considering the uniformity of the substrates, transfer processes, and contact metals across all the devices, the increased hysteresis window in the Nb-doped devices can be attributed to the trapping and detrapping of electrons at the doping site. At a negative gate voltage (V_gs_ = −60 V), carriers in the channel are entirely depleted, and trapped electrons lack the necessary thermal energy for capture. Conversely, with a positive gate voltage (V_gs_= 60 V), an abundance of free carriers quickly fills traps, resulting in a rightward shift in the threshold voltage and the establishment of clockwise hysteresis. Owing to the sensitivity of trap states to surface adsorbates in an air environment, we investigated the operational conditions of the transistors and the influence of environmental factors (Fig. S10). In Fig. 4d, pulse voltages of −30 V were applied to the gate to augment the current, whereas +30 V pulse voltages were employed to suppress the current. The current values were read at V_gs_ = 0 V after the pulses. For undoped WS_2_, the synaptic transistor switching ratio is merely 10 on the basis of the starting current. Remarkably, with minor Nb doping, the switching ratio increases by a factor of 100. P-type doping modulation enhances the switching ratio of the synaptic transistor via two main factors. First, doping introduces defects into the material, inducing a hysteresis curve in the FET by trapping and detrapping electrons. Second, p-type doping positively shifts the threshold voltage of the transistor, significantly enhancing the current ratio between high-resistance and low-resistance states at V_gs_ = 0 V. In summary, engineering hysteresis is achievable by increasing the Nb doping concentration, with 5% Nb-WS_2_ exhibiting the largest hysteresis loop, making it a suitable candidate for synaptic transistor construction in the context of TMDC materials.

**Fig. 5:**
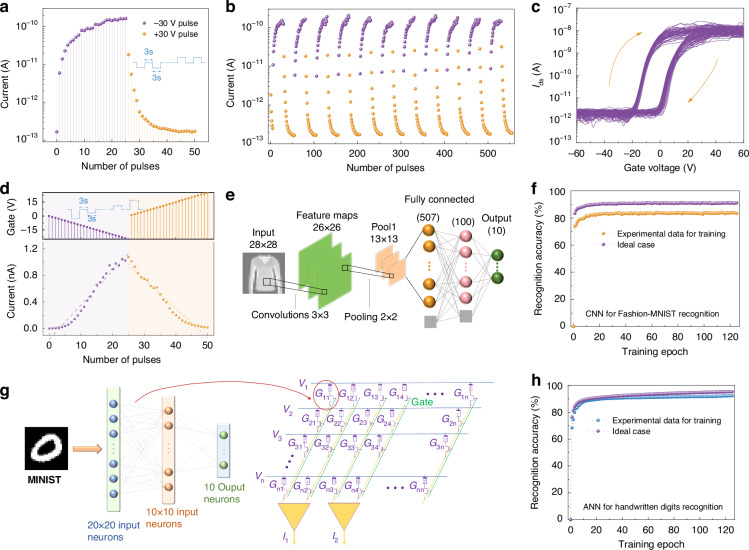
Performance of transistors simulating artificial synapses with 5% Nb-WS_2_ as the channel material. **a** Synaptic potentiation occurs during the first 25 pulses, whereas synaptic depression takes place during the subsequent 26th to 50th pulses, both of which are induced by voltage pulses of −30 V and 30 V. The current was assessed following the gate pulses (V_gs_ = 0 V). The inset is a diagram of the applied pulse. **b** Cyclic repetition of the potentiation and depression processes by alternating negative and positive pulses. **c** Device durability was assessed by conducting a 50-cycle transfer characteristic curve test. **d** Potentiation and depression behaviors are demonstrated by adjusting the gate bias. **e** Network structure of the five-level CNN for simulating pattern recognition. The evaluation is based on a Fashion-MNIST dataset. **f** Fashion-MNIST pattern recognition accuracy when potentiation/depression data are used. **g** Schematic diagrams depicting a three-layer ANN and simulated synaptic weight array for classifying MNIST handwritten digits. **h** The simulated accuracy of pattern recognition is depicted for synaptic transistors via 20 × 20-pixel handwritten digit image datasets

The synaptic behavior of the 5% Nb-WS_2_ transistor was further investigated. Figure 5a shows the long-term potentiation (LTP) and long-term depression (LTD) characteristics of the device during 25 potentiation (−30 V)–25 depression (+30 V) gate pulse trains. Following 25 cycles of negative gate voltage pulses, the transistor current consistently increased nearly 10^3^ times, increasing from 1.7 × 10^−13^ A to 1.5 × 10^−10^ A (Fig. 5a). With another 25 cycles of positive gate voltage pulses (+30 V), the current gradually receded to approximately 1.8 × 10^−13^ A. Thus, the Nb-WS_2_ transistor emulates both synaptic potentiation and depression responses. Even after 11 cycles of stimulation, the ratio between the high- and low-conductance states remains above 10^3^, and the consistent curve shape demonstrates the commendable reliability of this synaptic transistor (Fig. 5b). The Nb-WS_2_ synaptic transistor exhibited minimal baseline drift and low cycle-to-cycle variation, confirming its outstanding electrical durability. As depicted in Fig. 5c, throughout 50 repetitive transfer curve measurements, the hysteresis remains distinctly discernible. Furthermore, we demonstrated that dynamically controlling the gate bias during pulse training can significantly improve the linearity of the synaptic transistor. Figure 5d shows the current update with the stimulus bias gradually increasing from −1 to −25 V (in steps of −1 V), and the depression pulses are illustrated with a gradually decreasing bias from 1 to 25 V (in steps of 1 V).

To evaluate the neuromorphic computing performance of the Nb-WS_2_ transistor, we utilized performance data from single devices to simulate the behavior of the Nb-WS_2_ transistor arrays. We evaluated its convolution operation capabilities by constructing a five-layer convolutional neural network (CNN) for image recognition via the Fashion-MNIST dataset. As illustrated in Fig. 5e, the CNN structure comprises convolutional and pooling layers for feature extraction, along with fully connected layers for classification. By utilizing 28 × 28-pixel grayscale images as inputs, the CNN employs 3 × 3 kernels for convolutional operations. Subsequent to convolution, a pooling function facilitates downsampling, generating feature maps connected to the fully connected layer for image classification. In simulation experiments with the Nb-WS_2_ transistor array, the CNN achieved a final accuracy of 83.4% after 125 training epochs (see Fig. 5f). Moreover, within the framework of a three-layer artificial neural network (ANN) constructed with the Nb-WS_2_ transistor array, superior performance was observed in recognizing the MNIST handwritten digit dataset. Figure 5g illustrates the use of a multilayer perceptron neural network for performance benchmarking tests. A backpropagation algorithm was employed to assess the theoretical learning and recognition performance traits of artificial synaptic transistors fabricated from Nb-WS_2_. The ANN architecture comprises 400 input neurons (corresponding to 20 × 20 image pixels), 100 hidden layer neurons, and 10 output neurons (representing the 10 categories of Arabic numerals from 0 to 9). The diverse parameters for the Nb-WS_2_ artificial synaptic transistors were subsequently determined via the following equations:

1 $${{G}_{LTP}={\rm{B}}(1-{{\rm{e}}}^{(-\frac{P}{A})})+{G}_{{min }}}$$

2 $${G}_{{LTD}}=-{\rm{B}}(1-{e}^{\left(\frac{P-{P}_{max }}{A}\right)})+{G}_{max }$$

3 $${\rm{B}}=({G}_{{max }}-{G}_{{min }})/(1-{e}^{(-\frac{{P}_{{max }}}{A})})$$

where *G*_*LTP*_ and *G*_*LTD*_ represent the conductances for LTP and LTD, respectively. The parameters *G*_*max*_, *G*_*min*_, and *P*_*max*_ are derived from experimental data, indicating the maximum conductance, minimum conductance, and maximum number of pulses required for the device to switch between its minimum and maximum conductance states. Parameters A and B are the nonlinear fitting coefficients of LTP and LTD. In the context of handwritten digit dataset recognition, the device achieves a recognition accuracy of 92.3% after 125 training iterations, which is closely comparable to the 95.3% accuracy observed for the ideal synaptic device (Fig. 5h). These findings collectively underscore the robust endurance and stability of the synaptic transistor. This effectively mimics the repetitive learning progression exhibited by synapses, confirming that Nb-WS_2_ is a fitting material for the construction of artificial synaptic devices.

## Conclusion

In summary, we devised an effective methodology for achieving controlled Nb doping in monolayer 2D WS_2_. The doping concentration of Nb atoms can be tailored by manipulating the concentration of the liquid precursor. The uniformly distributed Nb atoms within WS_2_ establish a stable doping effect and electron characteristics, acting as trap centers to modulate the conductivity of WS_2_. By employing Nb-WS_2_ as the channel material, we fabricated gate-tunable synaptic transistors that effectively simulate synaptic potentiation, depression, and repetitive learning processes. The doping of Nb leads to a substantial increase in the switch ratio of the synaptic transistor, increasing it from 10 to 10^3^. In the recognition of the MNIST handwritten digit dataset, the Nb-WS_2_ synaptic transistor attains 92.30% accuracy after 125 training iterations. This work demonstrates controlled doping in 2D materials, offering versatile tuning of doping concentrations and providing a suitable platform for designing functional electronic devices, such as artificial synaptic transistors.

## Materials and methods

### CVD growth of WS_2_ and Nb-doped WS_2_

Si substrates measuring 15 mm × 15 mm with a 285 nm oxide layer underwent ultrasonic cleaning with acetone and isopropanol (IPA). The precursor solutions, which were composed of Nb(HC_2_O_4_)_5_·xH_2_O (99.99%, Aladdin) and Na_2_WO_4_·2H_2_O (99.99%, Aladdin) at atomic molar ratios, were spin-coated onto oxygen plasma treated substrates at 3000 rpm. Undoped WS_2_ and WS_2_ doped with nominal concentrations of 2% and 5% were grown while maintaining a constant concentration of 1.8 mg/ml to regulate the domain density. The Si substrates were positioned within the center of a quartz tube. Following a 20-minute Ar purge at a rate of 300 sccm, the furnace was heated to 875 °C within 30 min under the protection of 80 sccm Ar. A sufficient S source (200 mg, 99.95%, Aladdin) was placed upstream at the edge of the temperate zone. Upon reaching 780 °C in the main temperature zone, the S precursor was heated to 150 °C synchronously with the main temperature zone by a heating belt. After the growth process continued for 15 min, the furnace tube was naturally cooled to room temperature.

### Transfer of the sample onto a TEM Grid

The transmission samples were subjected to a polymer film-free method to minimize residues on the WS_2_ flake. Under microscopic guidance, the carbon film side of the copper mesh was gently positioned on the target sample. A drop of IPA was applied to the flake, which was allowed to evaporate naturally. The assembly was subsequently dried on a hot plate at 80 °C for 10 min to enhance the binding of WS_2_ to the carbon film. The silicon substrate, carrying the copper mesh, was immersed in a 1.5 mol/L KOH solution and etched at 60 °C for several hours until the substrate separated from the copper mesh. The copper mesh, retrieved with self-locking tweezers, was briefly soaked in deionized water to eliminate residual KOH and then subjected to a vacuum pump for 10 min to completely evaporate the adsorbed water.

### Materials characterization

Raman and PL measurements were conducted via a Raman spectrometer (Renishaw, U.K.) equipped with a 532 nm excitation laser. Prior to characterization, the system underwent calibration using standard Si with a peak at 520.5 ± 0.2 cm^−1^. The laser powers were maintained below 1 mW to prevent sample damage. The surface chemical state of the WS_2_ flakes was analyzed via Axis Ultra XPS (Kratos, UK) with an Al Kα X-ray source. The thickness of each WS_2_ flake was examined via AFM (Bruker, Dimension ICON). The atomic structure was investigated via ADF-STEM (FEI Titan ChemiSTEM) with an accelerating voltage of 200 kV. The SAED patterns were recorded with an FEI Tecnai F20 system.

### Device fabrication and measurement

The source and drain electrodes were defined through standard electron beam lithography. Electrodes were deposited via e-beam evaporation of 5 nm Cr followed by 10 nm Au. By employing a polymethyl methacrylate (PMMA)-assisted transfer method, WS_2_ sheets were transferred onto the prepared electrodes. Initially, PMMA (MicroChem, 950 A4) was coated on the substrate and spin-coated at 3000 rpm for 60 seconds. The samples were subsequently dried at 80 °C for 20 min and allowed to rest for 24 h to ensure the robustness of the polymer film. The samples were subsequently etched in a 1.5 mol/L KOH solution, after which the solution was heated to 60 °C to expedite the etching process. Upon separation of the PMMA film from the substrate, the film was picked up via tweezers and submerged in deionized water for 10 min to remove the KOH residue. This process was repeated three times. The meticulous transfer of WS_2_ nanosheets onto a substrate with predeposited electrodes was then executed under microscopic observation (see the details and schematic diagram in Fig. S6). The device measurements were conducted with a semiconductor characterization system (4200-SCS, Keithley, USA) using a vacuum probe station (10^−5^ Pa).

## Supplementary information

Supplementary Information
