# Supplementary material for: Atomic Nb-doping of WS2 for high-performance synaptic transistors in neuromorphic computing
Source: Microsyst Nanoeng. 2024 Sep 26;10:132. doi: 10.1038/s41378-024-00779-1 (PMC11427458; doi:10.1038/s41378-024-00779-1)
Supplement: Supplementary file 1 — Supplementary Information [file 41378_2024_779_MOESM1_ESM.docx]

Supplementary Information

Atomic Nb-Doping of WS_2_ for High-Performance Synaptic Transistors in Neuromorphic Computing

*Kejie Guan^1, 2^, Yinxiao Li^1, 3^, Lin Liu^1^, Fuqin Sun^1^, Yingyi Wang^1, 4^, Zhuo Zheng^1^, Weifan Zhou^1, 2^, Cheng Zhang^1, 5^, Zhengyang Cai*^1, 6^, Xiaowei Wang*^1, 2^, Simin Feng*^1, 2^, Ting Zhang*^1, 2, 7^*

^1^ i-Lab, Suzhou Institute of Nano-Tech and Nano-Bionics (SINANO), Chinese Academy of Sciences (CAS), Suzhou, Jiangsu, 215123, P. R. China

^2^ School of Nano-Tech and Nano-Bionics, University of Science and Technology of China, Hefei, Anhui, 230026, P. R. China

^3^ School of Electronic and Optical Engineering, Nanjing University of Science and Technology, Nanjing, Jiangsu, 210094, China

^4^ Department of Health and Environmental Sciences, Xi’an Jiaotong-Liverpool University, 111 Renai Road, Suzhou, Jiangsu, 215123, P. R. China

^5^ Jiangsu Key Laboratory of Micro and Nano Heat Fluid Flow Technology and Energy Application, School of Physical Science and Technology, Suzhou University of Science and Technology, Suzhou, Jiangsu, 215009, China

^6^ Department of Electronic Engineering, Jiangnan University, Wuxi, Jiangsu, 214122, P. R. China

^7^ Nano-X Vacuum Interconnected Workstation, Suzhou Institute of Nano-Tech & Nano-Bionics (SINANO), Chinese Academy of Sciences (CAS), Suzhou, Jiangsu, 215123, P. R. China.

*Corresponding author. E-mail: [tzhang2009@sinano.ac.cn](mailto:tzhang2009@sinano.ac.cn) (Ting Zhang); [smfeng2020@sinano.ac.cn](mailto:smfeng2020@sinano.ac.cn) (Simin Feng); [xwwang2022@sinano.ac.cn](mailto:xwwang2022@sinano.ac.cn) (Xiaowei Wang); [caizy@jiangnan.edu.cn](mailto:caizy@jiangnan.edu.cn) (Zhengyang Cai)


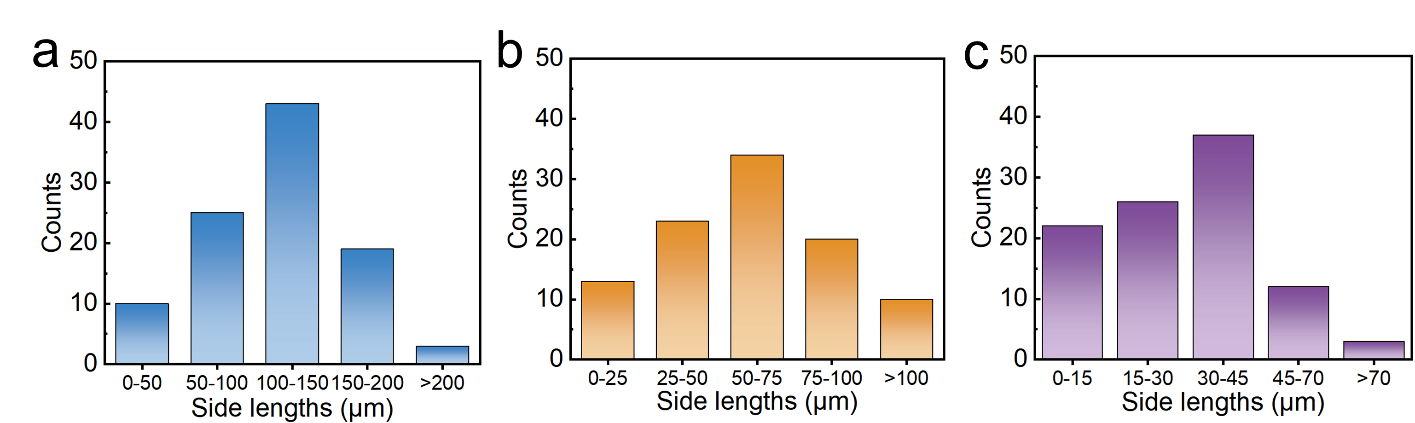


**Figure S1**. Side length statistics of triangular flakes under different doping concentrations: (a)Undoped WS_2_; (b) 2 % Nb-WS_2_; (c) 5 % Nb-WS_2_.

**
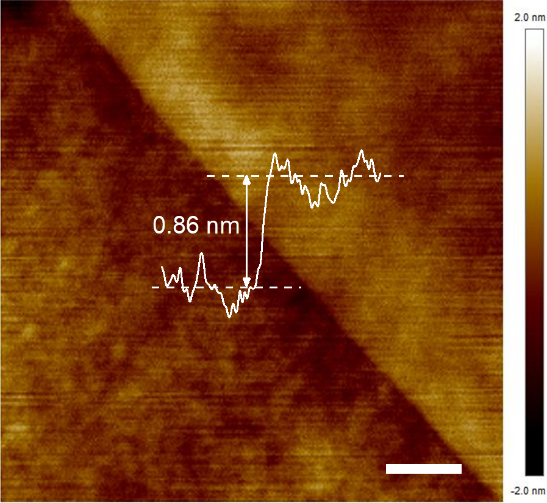
**

**Figure S2.** Typical AFM image of edge in WS_2_ sample. The height is measured to be 0.86 nm, confirming the monolayer of CVD prepared WS_2_. The scale bar is 10 nm.

**
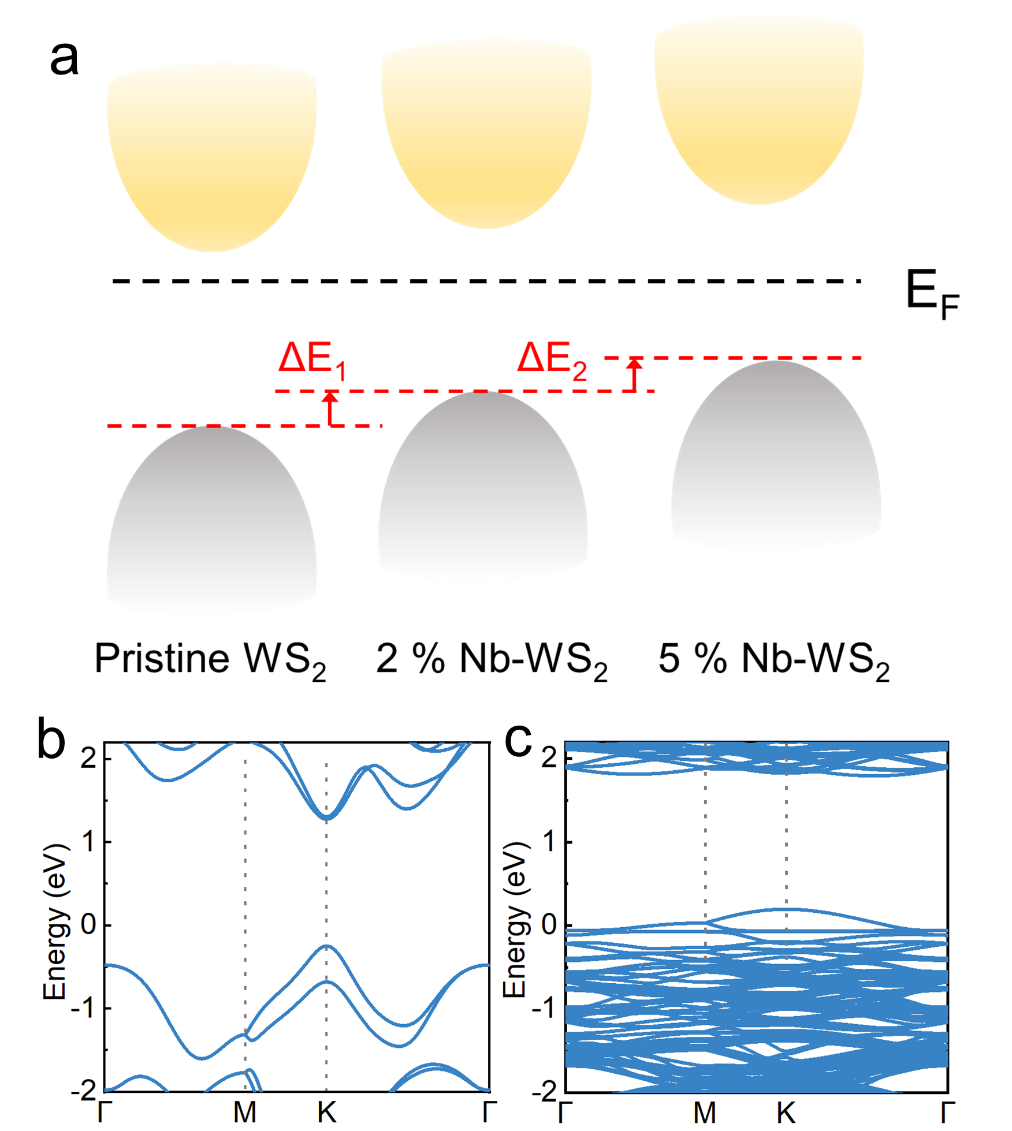
**

**Figure S3.** (a) Effects of atom doping on the electronic structure of WS_2_. Fermi levels are aligned. Energy band diagrams calculated by DFT for (b) WS_2_ and (c) Nb-WS_2_.

**Supplementary Note 1:**

The peaks of Trion and neutral exciton A, centered at ~1.95 and ~2.01 eV respectively, could be detected. The finite binding energy between the neutral exciton and the defect creates a bound-exciton peak X_B_ in the PL spectrum. ^[S1] [S2]^


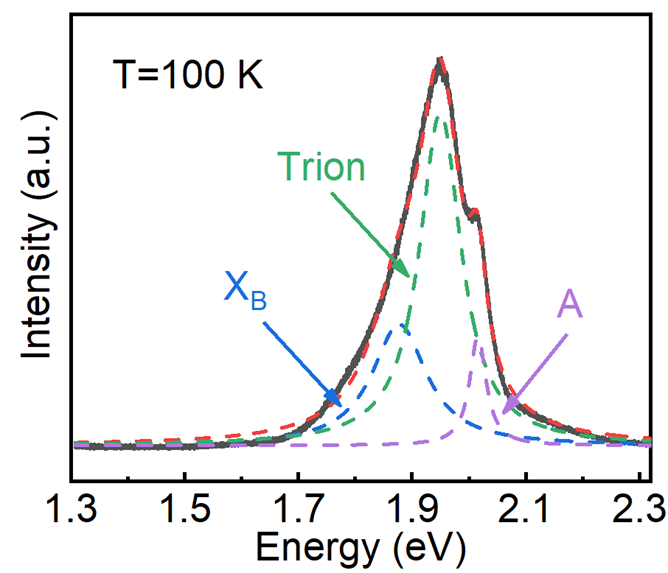


**Figure S4.** A typical curve fitting of 5 % Nb-WS_2_ PL spectrum with a Lorentzian at 100 K.

**Table S1** Binding energy for spin orbitals of different elements

| eV | Nb | | W | | | S | |
| --- | --- | --- | --- | --- | --- | --- | --- |
|  | Nb 3d_3/2_ | Nb 3d_5/2_ | W 4p_3/2_ | W 4f_5/2_ | W 4f_7/2_ | S 2p_1/2_ | S 2p_3/2_ |
| WS_2_ | N/A | N/A | 38.40 | 35.37 | 33.20 | 164.00 | 162.79 |
| 2% Nb-WS_2_ | 211.18 | 210.78 | 38.20 | 35.17 | 33.00 | 163.75 | 162.54 |
| 5% Nb-WS_2_ | 208.28 | 208.08 | 38.00 | 34.97 | 32.80 | 163.50 | 162.29 |


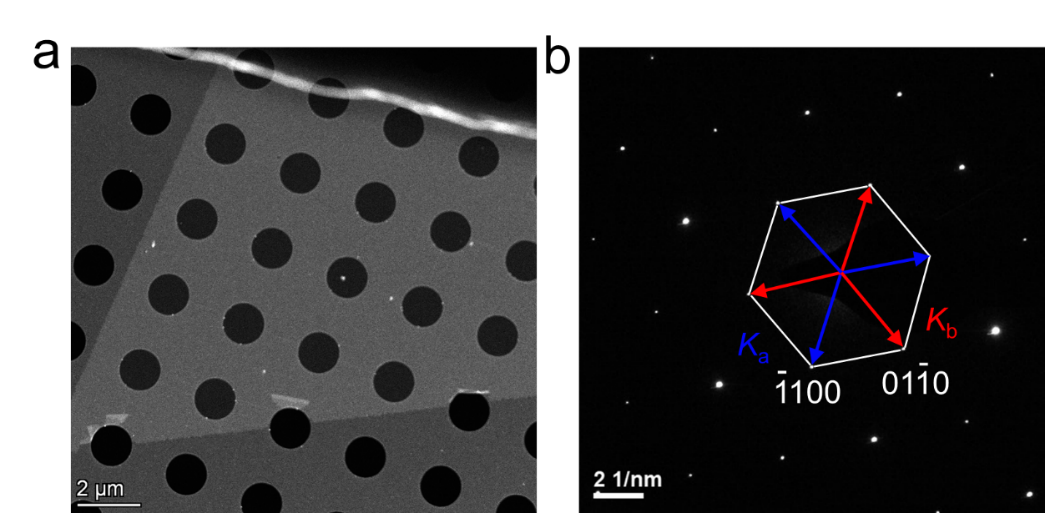


**Figure S5.** (a) Low magnification photographs in the TEM field of view. (b) SAED pattern of WS_2_ flake.

**Supplementary Note 2:**

**Transfer Process of Nb-WS_2_  onto Metal Electrodes:**

(i) A holed blue adhesive tape was attached to the spin-coated PMMA film on the Nb-WS_2_. This assembly (blue tape/PMMA/Nb-WS_2_/SiO_2_/Si) was floated in a 1.5 mol/L KOH solution for 3-5 hours, leading to the separation of the blue tape/PMMA/Nb-WS_2_ from the SiO_2_/Si growth substrate.

(ii) The blue tape/PMMA/Nb-WS_2_ structure, now floating on the KOH solution, was carefully lifted out using a tweezer due to the self-supporting nature of the blue tape. This structure was then floated in deionized water for 10 minutes to remove any residual KOH, and this washing process was repeated three times.

(iii) The cleaned structure was adhered to a PDMS-coated slide, ensuring the Nb-WS_2_ was exposed to air without contacting the PDMS. The blue tape was then removed, allowing the PDMS to support the PMMA/Nb-WS_2_ structure.

(iv) The PDMS/PMMA/Nb-WS_2_ structure, now transparent and adhered to a glass slide, was aligned with the fixed metal electrodes using a low-dimensional material transfer platform (Metatest Corporation, Nanjing, China). The height of the glass slide was gradually lowered until the Nb-WS_2_ made full contact with the metal electrodes.

(v) The entire assembly was heated to 110 °C to reduce the adhesive strength of the PDMS at its glass transition temperature, facilitating its separation from the PMMA. Consequently, the PMMA/Nb-WS_2_/metal electrode/SiO_2_/Si structure was obtained.

(vi) Finally, this structure was immersed in 40 °C acetone for 10 minutes to dissolve and remove the PMMA, leaving the Nb-WS_2_ in contact with the metal electrodes.


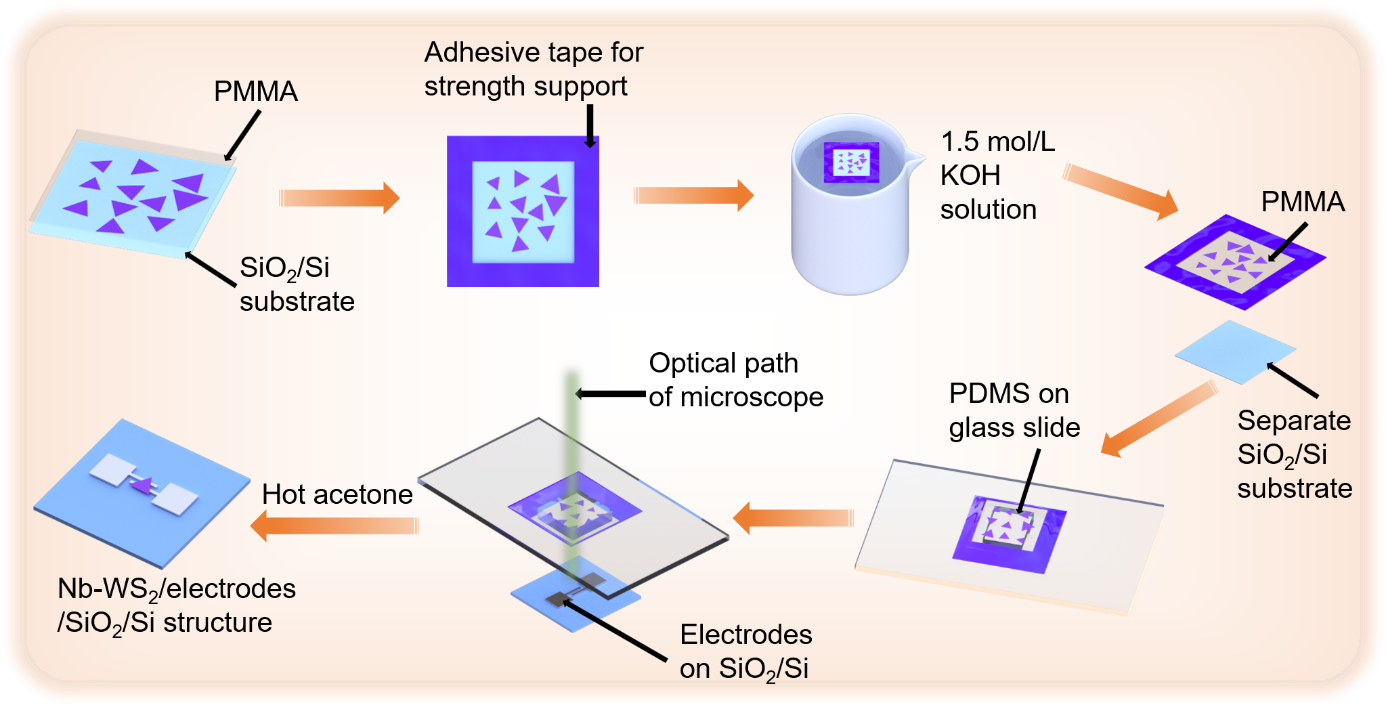


**Figure S6**. Schematic diagram of material transfer process.


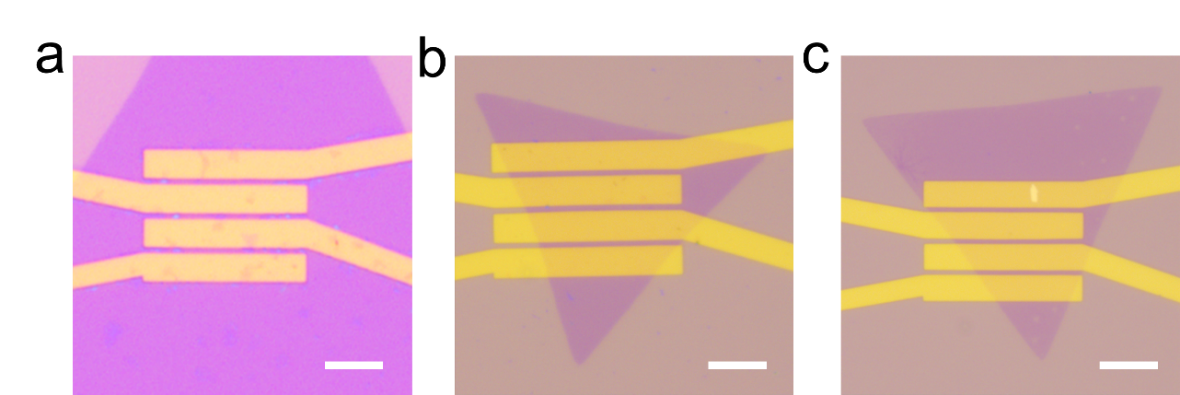


**Figure S7.** Typical WS_2_ and Nb-WS_2_ FET devices with channel lengths of 1 µm. The flakes were transferred by wet chemical etching to fabricate the devices. Scale bar: 10 μm.

**Supplementary Note 4:**

According to definition about device parameters, our devices have a fixed channel length of 1 μm, while the channel width varies based on the geometry of the Nb-WS_2_ flakes. ^[S3]^ As reported in the literature, the relationship between *I_ds_*_​_ and the geometric parameters of the channel material is as follows:

$$I_{ds}=\frac{WC_{i}}{L}\mu(V_{gs}-V_{t}-\frac{V_{t}}{2})V_{ds}$$

where *W* is the channel width, *L* is the channel length, *μ* is the mobility, *V_t_* is the threshold voltage and *C_i_* is the capacitance of the 285-nm-thick thermally grown SiO_2_ film. ^[S4][S5]^ It indicates that devices with different channel widths may exhibit varying on-state and off-state currents, but the on/off ratio remains unaffected.

To address the reviewer's concern, we investigated the performance of devices with different channel widths to understand the impact of these variations. We transferred a flake with a precursor concentration of 2% Nb doping and fabricated devices with channel widths of 15 μm, 24 μm, and 27 μm, as shown in Figure S3a. Our experimental results are summarized below:

Figure S3b demonstrates that devices with different channel widths exhibit varying drain currents. The transfer characteristic curves in Figure S3c show that devices with channel widths of 24 μm and 27 μm have almost identical on-state and off-state currents, measured at 5.05×10^−8^ A and 2.47×10^−13^ A, respectively. Conversely, the device with a 15 μm channel width shows lower on-state and off-state currents, recorded at 1.90×10^−8^ A and 1.78×10^−8^ A, respectively. Despite the differences in current levels, the on/off ratios of the devices with 24 μm and 15 μm channel widths are nearly identical, measured at 1.12×10^5^ and 1.06×10^5^, respectively. Our experimental results confirm that while the absolute current levels vary with the channel width, the on/off ratio remains consistent across devices, which aligns with theoretical predictions.


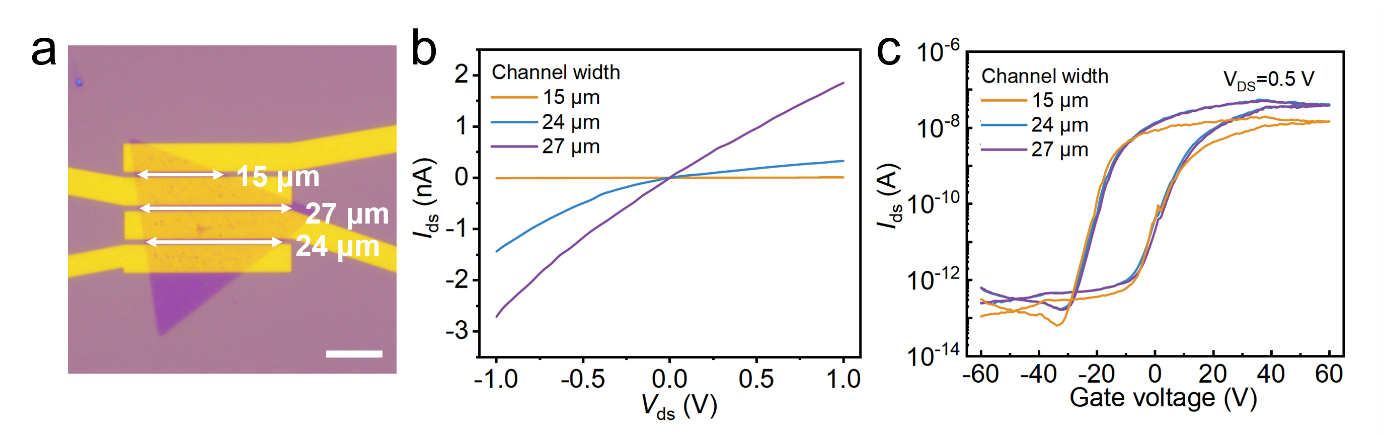


**Figure S8**. Performance of devices with different channel widths. (a) Optical image of devices with different channel widths. (b) *I-V* curves and (c) transfer characteristic curves of devices with 15 μm, 24 μm and 27 μm channel widths.

**Supplementary Note 5:**

As illustrated in Figure S9a, the resistance of the channel material escalated to approximately 10^12^ Ω at higher doping level of 7 % nominal Nb concentration, clearly indicating a significant reduction in electron transport capabilities. This increase in resistance suggests that excessive doping adversely affects the material’s conductive properties. Furthermore, the transfer characteristic curves depicted in Figure S9b demonstrate that devices with a nominal Nb concentration of 7 % exhibited a significantly reduced on/off ratio to about 100. This substantial decline in switching performance severely impacted the device's ability to achieve LTP and LTD, which are critical for mimicking synaptic behaviors in neuromorphic applications. When these devices were employed in an artificial neural network for the recognition of the MNIST handwritten digit dataset, the accuracy achieved was only 81.20 % after 125 training iterations. This performance is notably lower compared to devices with lower doping concentrations.


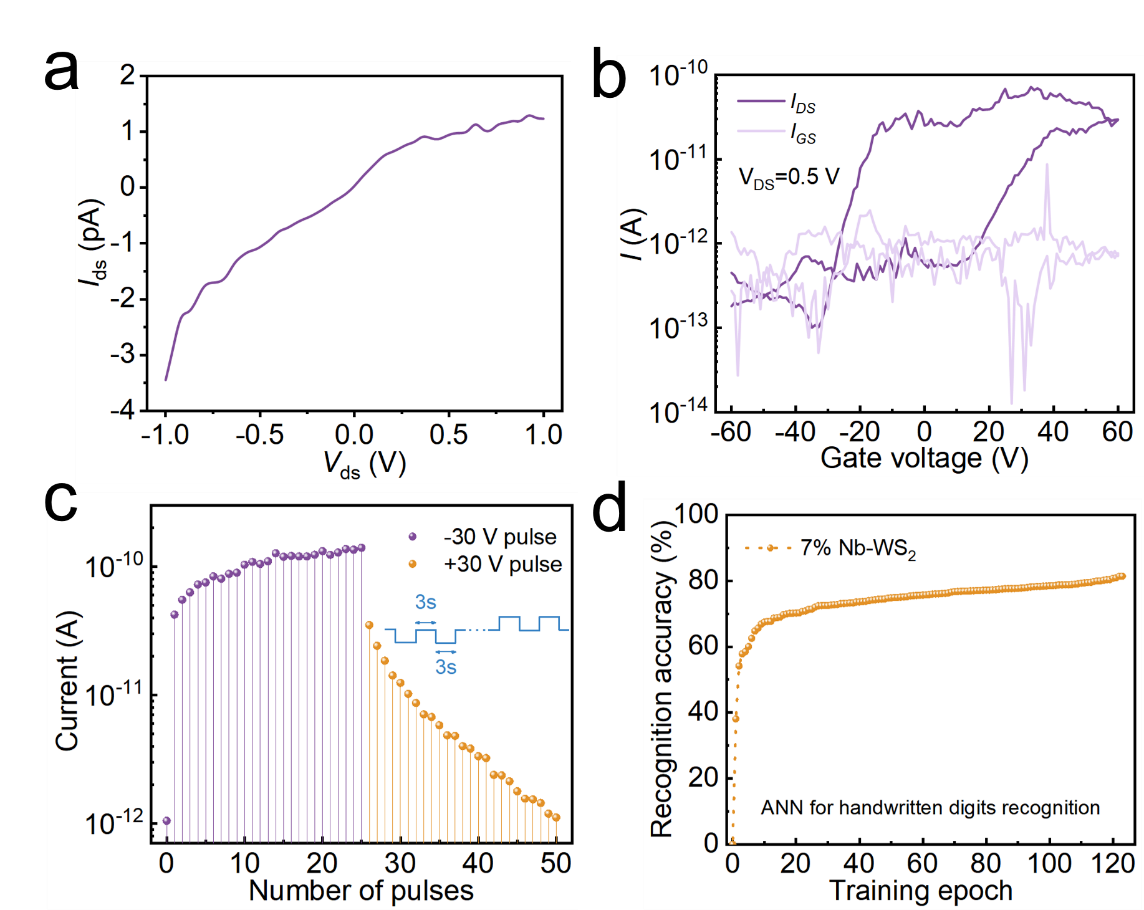


**Figure S9.** Devices with a nominal Nb concentration of 7 %. (a) *I-V* curve and (b) transfer characteristic curve of the device. (c) Synaptic potentiation and depression induced by voltage pulses of -30 V and 30 V. (d) Simulated accuracy of pattern recognition for synaptic transistor.

**Supplementary Note 6:**

Long-term stability tests were performed on the Nb-WS_2_ transistors to evaluate their reliability under continuous operation. As depicted in Figure R4, 5 % Nb-WS_2_ transistors in vacuum exhibited an on/off ratio of approximately 5×10^3^. After storing the device in vacuum for 15 days, we re-evaluated its performance. The results showed minimal changes in the transfer characteristic curves' on/off ratio and threshold voltage, indicating high stability of the transistor under operational conditions.

To assess the impact of environmental factors such as temperature and humidity, the transfer characteristics of the 5 % Nb-WS_2_ transistor was measured under vacuum and air conditions. In the air environment, the temperature was maintained at 25 ℃ and the humidity at 60 %. Under vacuum conditions, a higher current switching ratio was observed due to fewer interface trap states and reduced surface scattering. In contrast, in the air environment, surface adsorbates increased interface scattering and carrier recombination, resulting in a decreased current switching ratio. Regarding threshold voltage, a lower threshold voltage was recorded under vacuum conditions due to the lack of interaction between oxygen and water molecules with the Nb-WS_2_ surface. This reduction in surface adsorbates and interface trap states led to less charge capture and scattering. Due to the constantly changing temperature and humidity in the air, this will have an impact on device performance. It is evident that our devices are suitable for use after proper packaging, such as BN and PMMA, to mitigate these environmental impacts.


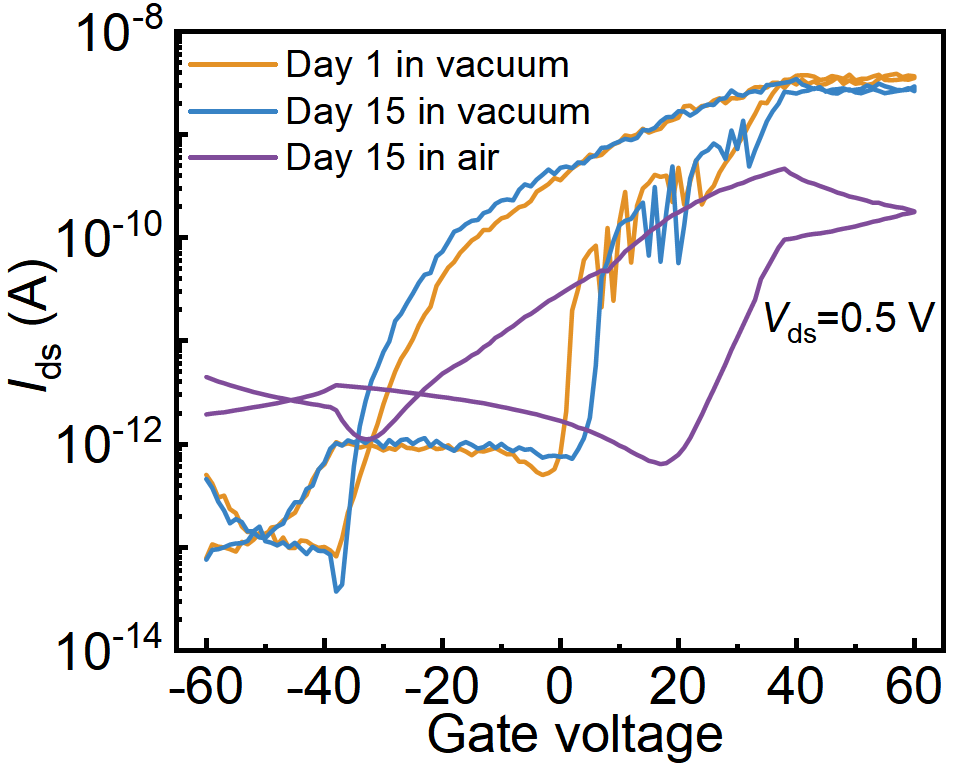


**Figure S10**. Transfer characteristic curves of devices in vacuum and air environments.

**References**

[S1] Mak, K.F.; He, K.L.; Lee, C.; Lee, G.H.; Hone, J.; Heinz, T.F.; Shan, J. Tightly bound trions in monolayer MoS_2_. *Nat. Mater.* **12,** 207-211, (2013).

[S2] Linhart, L.; Paur, M.; Smejkal, V.; Burgdörfer, J.; Mueller, T. Localized Intervalley Defect Excitons as Single-Photon Emitters in WSe_2_. *Phys. Rev. Lett.* **123,** 146401, (2019).

[S3] Z.H. Cheng, C.S. Pang, P.Q. Wang, S.T. Le, Y.Q. Wu, D. Shahrjerdi, How to report and benchmark emerging field-effect transistors, *Nat. Electron.* **5,** 416-423, (2022).

[S4] S.H. Bae, H.J. Ryoo, J.H. Yang, Y.H. Kim, C.S. Hwang, S.M. Yoon, Influence of Reduction in Effective Channel Length on Device Operations of In-Ga-Zn-O Thin-Film Transistors with Variations in Channel Compositions, *Ieee T. Electron. De.* **68,** 6159-6165 (2021).

[S5] S.J. Kang, M. Noh, D.S. Park, H.J. Kim, S.Y. Kim, B.W. Koo, I.N. Kang, C.N. Whang, Geometric effect of channel on device performance in pentacene thin-film transistor, *Jpn. J. Appl. Phys.* **43,** 7718-7721 (2004).
